# Comparing stimulus-evoked and spontaneous response of the face-selective multi-units in the human posterior fusiform gyrus

**DOI:** 10.1093/nc/niab033

**Published:** 2021-10-16

**Authors:** Rina Schwartz, Camille Rozier, Tal Seidel Malkinson, Katia Lehongre, Claude Adam, Virginie Lambrecq, Vincent Navarro, Lionel Naccache, Vadim Axelrod

**Affiliations:** The Gonda Multidisciplinary Brain Research Center, Bar Ilan University, Ramat Gan 52900, Israel; Department of Psychology, Bar-Ilan University, Ramat Gan 5290002, Israel; Institut National de la Santé et de la Recherche Médicale Unité 1127, Centre National de la Recherche Scientifique Unité Mixte de Recherche (UMR) 7225, Université Pierre-et-Marie-Curie Univ Paris 06 UMR S 1127, Institut du Cerveau et de la Moelle Épinière ICM, Paris 75013, France; Institut National de la Santé et de la Recherche Médicale Unité 1127, Centre National de la Recherche Scientifique Unité Mixte de Recherche (UMR) 7225, Université Pierre-et-Marie-Curie Univ Paris 06 UMR S 1127, Institut du Cerveau et de la Moelle Épinière ICM, Paris 75013, France; Institut National de la Santé et de la Recherche Médicale Unité 1127, Centre National de la Recherche Scientifique Unité Mixte de Recherche (UMR) 7225, Université Pierre-et-Marie-Curie Univ Paris 06 UMR S 1127, Institut du Cerveau et de la Moelle Épinière ICM, Paris 75013, France; Centre de NeuroImagerie de Recherche­CENIR, Institute of Brain and Spine, UMRS 1127, CNRS UMR 7225, Pitié­Salpêtriere Hospital, 47­83 boulevard de l’Hôpital, Paris 75013, France; Neurology Department, AP−HP, GH Pitie−Salpêtrière−Charles Foix, Epilepsy Unit, 47-83 boulevard de l’Hôpital, Paris 75013, France; Institut National de la Santé et de la Recherche Médicale Unité 1127, Centre National de la Recherche Scientifique Unité Mixte de Recherche (UMR) 7225, Université Pierre-et-Marie-Curie Univ Paris 06 UMR S 1127, Institut du Cerveau et de la Moelle Épinière ICM, Paris 75013, France; Department of Neurophysiology, AP-HP, Groupe Hospitalier Pitié-Salpêtrière, 47-83 boulevard de l’Hôpital, Paris 75013, France; Sorbonne Université, UMR S1127, 47-83 boulevard de l’Hôpital, Paris 75013, France; Institut National de la Santé et de la Recherche Médicale Unité 1127, Centre National de la Recherche Scientifique Unité Mixte de Recherche (UMR) 7225, Université Pierre-et-Marie-Curie Univ Paris 06 UMR S 1127, Institut du Cerveau et de la Moelle Épinière ICM, Paris 75013, France; Neurology Department, AP−HP, GH Pitie−Salpêtrière−Charles Foix, Epilepsy Unit, 47-83 boulevard de l’Hôpital, Paris 75013, France; Sorbonne Université, UMR S1127, 47-83 boulevard de l’Hôpital, Paris 75013, France; Institut National de la Santé et de la Recherche Médicale Unité 1127, Centre National de la Recherche Scientifique Unité Mixte de Recherche (UMR) 7225, Université Pierre-et-Marie-Curie Univ Paris 06 UMR S 1127, Institut du Cerveau et de la Moelle Épinière ICM, Paris 75013, France; Department of Neurophysiology, AP-HP, Groupe Hospitalier Pitié-Salpêtrière, 47-83 boulevard de l’Hôpital, Paris 75013, France; The Gonda Multidisciplinary Brain Research Center, Bar Ilan University, Ramat Gan 52900, Israel

**Keywords:** spontaneous activity, stimulus-evoked activity, face-selective activity, conscious awareness, multi-unit recording in humans, Fusiform Face Area (FFA)

## Abstract

The stimulus-evoked neural response is a widely explored phenomenon. Conscious awareness is associated in many cases with the corresponding selective stimulus-evoked response. For example, conscious awareness of a face stimulus is associated with or accompanied by stimulus-evoked activity in the fusiform face area (FFA). In addition to the stimulus-evoked response, spontaneous (i.e. task-unrelated) activity in the brain is also abundant. Notably, spontaneous activity is considered unconscious. For example, spontaneous activity in the FFA is not associated with conscious awareness of a face. The question is: what is the difference at the neural level between stimulus-evoked activity in a case that this activity is associated with conscious awareness of some content (e.g. activity in the FFA in response to fully visible face stimuli) and spontaneous activity in that same region of the brain? To answer this question, in the present study, we had a rare opportunity to record two face-selective multi-units in the vicinity of the FFA in a human patient. We compared multi-unit face-selective task-evoked activity with spontaneous prestimulus and a resting-state activity. We found that when activity was examined over relatively long temporal windows (e.g. 100–200 ms), face-selective stimulus-evoked firing in the recorded multi-units was much higher than the spontaneous activity. In contrast, when activity was examined over relatively short windows, we found many cases of high firing rates within the spontaneous activity that were comparable to stimulus-evoked activity. Our results thus indicate that the sustained activity is what might differentiate between stimulus-evoked activity that is associated with conscious awareness and spontaneous activity.

**Highlights**

- Face-selective stimulus-evoked vs. spontaneous multi-unit activity in human brain is examined
- Long periods of interest: higher conscious stimulus-evoked than unconscious spontaneous activity
- Short periods of interest: comparable conscious stimulus-evoked and unconscious spontaneous
- Sustained activity is associated with conscious evoked but not unconscious spontaneous response

## Introduction

The stimulus-evoked neural response—an increase of neural activity following stimulus presentation—is probably the most robust and well-explored phenomenon related to neural processing (Friston 2005). This phenomenon is universal and can be observed across a variety of brain regions, modalities (e.g. visual and auditory), and species. The stimulus-evoked neural response can additionally be detected using various recording and imaging methods [e.g. intracranial and scalp electrophysiological recordings, functional magnetic resonance imaging (fMRI)]. Conscious awareness is associated in many cases with a corresponding selective stimulus-evoked response. For example, conscious awareness about a face stimulus is likely always associated with activity in the fusiform face area (FFA) (Kanwisher 2017). Note that there is still no agreement regarding whether stimulus-evoked responses in specific regions (such as the FFA) contribute directly to conscious awareness (Block 2007; Boly *et al.* 2017) or if conscious awareness is achieved, for example, by global neuronal workspace integration (Dehaene and Naccache 2001; Mashour *et al.* 2020).

An additional omnipresent phenomenon is the spontaneous (or ongoing, intrinsic, resting-state) activity—the type of neural activity that occurs constantly in the background and that is not triggered by any stimuli (Raichle *et al.* 2001). One of the first demonstrations of spontaneous activity was the electroencephalogram (EEG) recordings done by Hans Berger (Berger 1929). Renewed interest in spontaneous activity was sparked by the pioneering works of Arieli, Grinvald, Tsodyks and colleagues (Arieli *et al.* 1995, 1996; Tsodyks *et al.* 1999) conducted in the early visual cortex in anesthetized cats. Examination of co-variation of spontaneous activity from two or more neural sources gave rise to the resting-state functional connectivity approach—one of the most prominent research directions today (Biswal *et al.* 1995; Damoiseaux *et al.* 2006; Mantini *et al.* 2007; Bullmore and Sporns 2009; Yeo *et al.* 2011; Lurie *et al.* 2019; Marron *et al.* 2020). The relationship between spontaneous activity and conscious awareness is complex. In certain scenarios, spontaneous activity has been shown to modulate cognitive behavior in domains such as visual perception (Hesselmann *et al.*2008b; Hahamy *et al.* 2020), creative thinking (Broday-Dvir and Malach 2021), and volitional decision-making (Schurger *et al.* 2012). Spontaneous activity has been also suggested to play a role in consciousness (Northoff and Lamme 2020), for example, by integrating information across brain regions (He and Raichle 2009) or across brain regions (i.e. space) and time (Northoff 2013; Northoff and Huang 2017). However, the spontaneous neural activity per se is unconscious because a person does not know when the spontaneous wave is at its minimum or maximum in a given brain region. For example, while the spontaneous activity fluctuates several times per minute in the FFA and the parahippocampal place area (PPA) high-level visual areas (Nir *et al.* 2006), we do not have a constantly alternating experience of a face and a scene.

The question is what is the difference at the neural level between stimulus-evoked activity in a case that this activity is associated with conscious awareness of some content and spontaneous activity? In other words, are there any properties of the neural signal that may explain whether we are consciously aware of the stimulus or not? Surprisingly, to date, there have been few studies that compared stimulus-evoked responses and spontaneous activity, and they do not provide sufficient answers. One prominent functional MRI (fMRI) study (Nir *et al.* 2006) demonstrated that the amplitude of spontaneous fluctuations in the FFA and the PPA was comparable to the stimulus-evoked responses. However, because fMRI measures hemodynamic activity, which is only an indirect measure of a neural response (Logothetis *et al.* 2001), it is challenging to extrapolate this result to neural activity. Two additional studies compared single-unit stimulus-evoked and spontaneous activity in the cat primary visual areas (Arieli *et al.* 1995) and the human primary auditory cortex (Nir *et al.* 2008). Both of these studies showed that stimulus-evoked activity was associated with higher firing rates compared to spontaneous activity. But these studies did not focus on the question of whether periods of high firing rates can still be found in spontaneous activity. In addition, investigations in these studies have been conducted in primary cortices, regions that are unlikely to contribute directly to conscious awareness (Dehaene *et al.* 2006; Watanabe *et al.* 2011).

**Figure 1.**
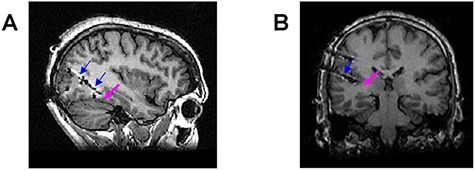
Anatomical image of the patients with the location of the electrodes. Blue arrows point to the implanted depth electrode. Magenta arrow points to the approximate location of the microwires. (A) Anatomical image of the patient with the micro-wires implanted in the posterior fusiform gyrus. (B) Anatomical image of the control patient with the micro-wires implanted in the planum temporale.

The present intracranial study was conducted with patients who underwent clinical monitoring for epileptic seizures. We had a rare opportunity to record multi-unit activity (MUA) of two strongly face-selective multi-units located in the posterior fusiform gyrus of one patient (Fig. 1A). As we reported previously (Axelrod *et al.* 2019), the multi-units were located in the vicinity of the FFA. As the control region, activity in the planum temporale (i.e. auditory cortex) was recorded in an additional patient (Fig. 1B). The present investigation included a task-based experiment with face stimuli as well as a resting-state session. This setup and design allowed us to compare electrophysiological face-selective stimulus-evoked responses, spontaneous activity that preceded face stimulus (i.e. fixation period), and spontaneous activity during resting-state session in a high-level visual area of a human brain.

## Methods

Detailed information about the patient with face-selective multi-units, recording procedures, and stimulus-evoked paradigm have been provided in detail in supplementary materials of our previous publication (Axelrod *et al.* 2019). Here, we provide the most essential information.

### Information about patients

The face-selective activity was recorded in a 26-year-old female subject (i.e. patient). For clinical epilepsy seizure monitoring, the patient was implanted with depth electrodes in the right occipital and temporal lobes. The experiment with the control region was conducted in a 21-year-old male patient for whom depth electrodes were implanted in the left temporal, parietal, and frontal lobes. For both patients, no epileptic activity was found in the regions of interest of the present investigation (i.e. right posterior fusiform gyrus in the patient with face-selective multi-units and left planum temporale in the control patient). Note that the location of the electrodes in such a procedure is determined solely by clinical criteria. The data were recorded during the subjects’ hospitalization in the Epilepsy ward of Pitié-Salpétrière Hospital in accordance with approval and guidelines of the local ethics committee (CPP Paris VI, INSERM C11-16), with the same clinical setup as the previous intracranial studies conducted in Pitié-Salpétrière Hospital (El Karoui *et al.* 2014; Babo-Rebelo *et al.* 2016; Corlier *et al.* 2016; Chammat *et al.* 2017).

### Recording setup

Depth platinum macro-electrodes (AdTech, Wisconsin) of the Behnke-Fried type 5 were used for stereotactic EEG recording. The electrodes of interest for the present study were implanted in the right posterior fusiform gyrus (Fig. 1A; the patient with face-selective multi-units) and the left planum temporale (Fig. 1B; the control patient). Eight platinum–iridium microwires were located at the tip of this electrode (protruded about 5 mm beyond the macro-electrode). Note, that there is no way to determine the exact location of microwires in human electrophysiological recordings (Self *et al.* 2016); therefore, we describe only the approximate location of the microwires (Fig. 1, magenta arrow). In our previous study (Axelrod *et al.* 2019), we established that the microwires that recorded the face-selective activity were in the vicinity of the FFA. The microwire recording (the focus of this paper) was conducted using the Atlas recording system (Neuralynx Inc., Tucson, AZ). The microwire recording sampling rate was 32 kHz and the online band-pass filter was 0.1–4000 Hz.

### Experimental paradigms

The experiment was conducted in a quiet room in the hospital ward. Stimuli were projected on a Dell Precision M4600 laptop (15.6-inch display, 1366 × 768 resolution) using MATLAB with Psychtoolbox-3 (Brainard 1997). The patients were sitting ∼50 cm from the monitor.

### Resting-state session

The standard resting-state paradigm was used (Fox *et al.* 2006). The duration of the resting-state session was 5 minutes and 50 seconds. During this time, the patients remained with their eyes closed. They were also asked to not think about anything specific and in particular to not imagine anything. No formal questionnaires regarding imagery were issued after the resting-state session.

### Stimulus-evoked experiment

The experiment included color images of faces (120 trials), city views (hereinafter referred as “scenes”; 120 trials), and everyday objects (40 trials). Two types of faces were presented, familiar and unfamiliar. Faces familiar to patients were famous faces (French public figures), and faces not familiar to the patient were non-French public figures. Familiar and unfamiliar scenes were presented as well. Familiar scenes were city views of Paris, and unfamiliar scenes were views of cities from outside of France. The size of the stimuli was about 11° visual angles. Each stimulus was shown for 1 second and was preceded by a 1.2-second fixation. The behavioral task was to indicate whether the image was familiar or not. The main motivation behind this study was to compare the MUA neural properties of face-selective stimulus-evoked response and spontaneous activity. Because we were interested in the neural response to the stimulus category, we combined familiar and unfamiliar stimuli, resulting in two conditions: “faces” and “scenes.” The scenes condition was only used only to independently establish face-selectivity. Activity preceding the faces stimuli was analyzed as well (referred to below as “prestimulus activity”). The objects category was not included in the analysis because an additional control condition did not contribute any new information and because there were many fewer trials with objects stimuli, compared to the number of trials in the faces and scenes conditions.

### Data analysis

Data analysis was done in MATLAB using FieldTrip (Oostenveld *et al.* 2011), Chronux toolbox (http://chronux.org/) (Mitra 2007), wave_clus (Quiroga *et al.* 2004), and Matlab Offline Spike Sorting (https://github.com/JoaquinMansilla/Spike-Sorter) toolboxes as well as custom code (Axelrod 2014). Spike detection and sorting were executed using a standard procedure for analyzing MUA recordings in humans (Quiroga *et al.* 2005; Reddy *et al.* 2015; Kornblith *et al.* 2017). Before performing the MUA analysis, the Local Field Potential (LFP) signals of the microwires were inspected to minimize the possibility of interference from local epileptic activity. No local epileptic activity was found. Spike detection and spike sorting were done using wave_clus toolbox. The average of the cluster did not exceed 50 μV in either of the units, and they were therefore classified as MUA. To validate the results of spike-detection and spike-sorting, we conducted two types of control analysis. One validation approach was to use our main analysis pipeline (wave_clus toolbox) to conduct spike-detection with three different threshold values (stdmin = 4, stdmin = 4.5 and stdmin = 5). Another validation approach was to use a different software package for spike-detection and spike-sorting (Matlab Offline Spike Sorting toolbox and custom procedure). The results we obtained using both validation approaches were similar to the results using our main analysis pipeline.

**Figure 2.**
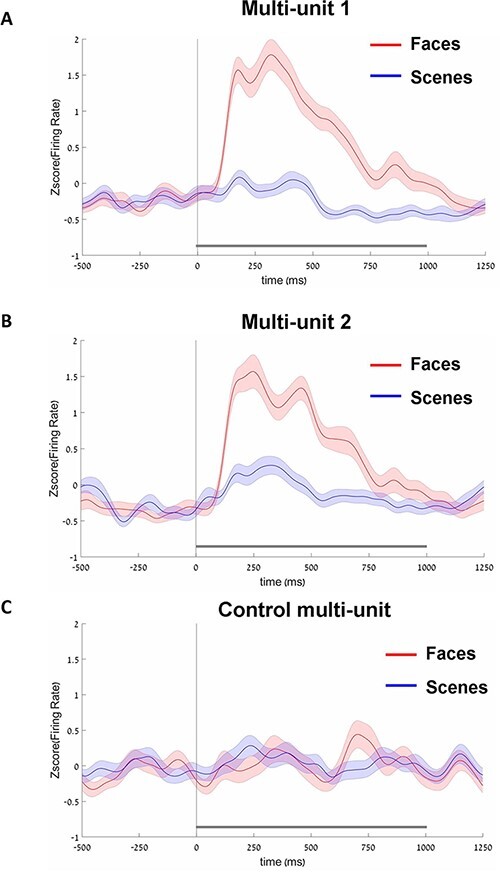
Average firing-rate time courses for the faces and scenes conditions. (A) Results in multi-unit 1. (B) Results in multi-unit 2. (C) Results in control multi-unit. Note the robust stimulus-evoked response for faces in multi-units 1 and 2 but not in the control unit. Error bars indicate standard error of mean (SEM). Note, that this analysis was conducted using independent, second part of the data.

**Figure 3.**
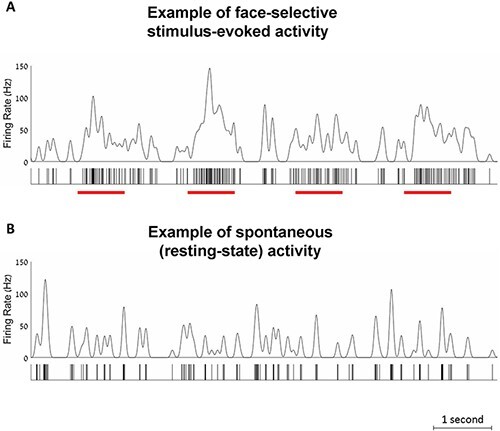
Examples of instantaneous firing rates over time, for multi-unit 1. Below each graph is a raster plot of the spikes recorded during that time. (A) Example of stimulus-evoked activity. The times that face stimuli were presented are indicated by red bars below the raster plot. (B) Example of resting-state activity. Note that strong activity was found even in spontaneous activity, but this was not sustained as it was during stimulus-evoked activity.

**Figure 4.**
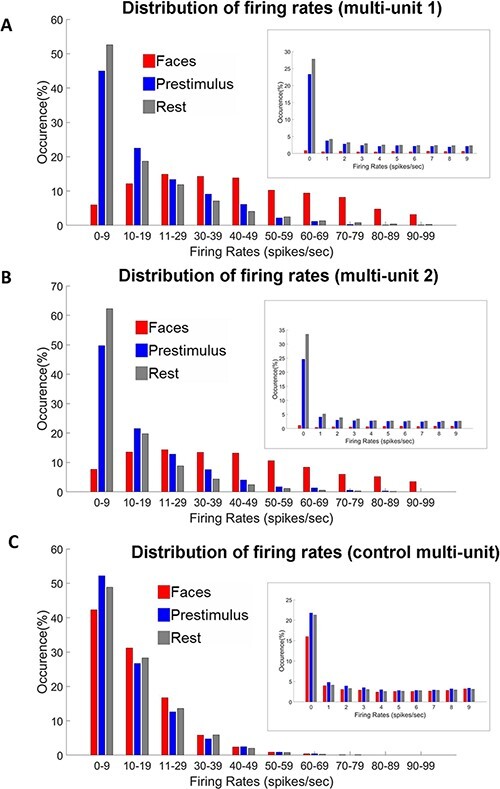
Distribution of stimulus-evoked and spontaneous firing rates. The larger histogram shows the distribution of firing rates from 0 to 100 spikes per second. For better visibility, the histograms were trimmed at 100 spikes per second. The histogram in the inset zooms in on the range from 0 to 9 spikes per second. The y axis shows the relative frequency of each range of firing rates. The main finding for both multi-units was that the low firing rates were abundant in the resting state (gray bars) but were uncommon in the faces condition (red bars); in contrast, the high firing rates were abundant in the faces condition but were almost absent in the resting state. (A) Results in multi-unit 1. Beyond 100 spikes per second were 4.1% of faces, 0% of prestimulus, and 0.4% of the resting-state firing rates. (B) Results in multi-unit 2. Beyond 100 spikes per second were 5.5% of faces, 0.1% of prestimulus, and 0.1% of the resting-state firing rates. (C) Results in control multi-unit. Beyond 100 spikes per second, there were 0% of faces, 0% of prestimulus, and 0% of the resting-state firing rates.

**Figure 5.**
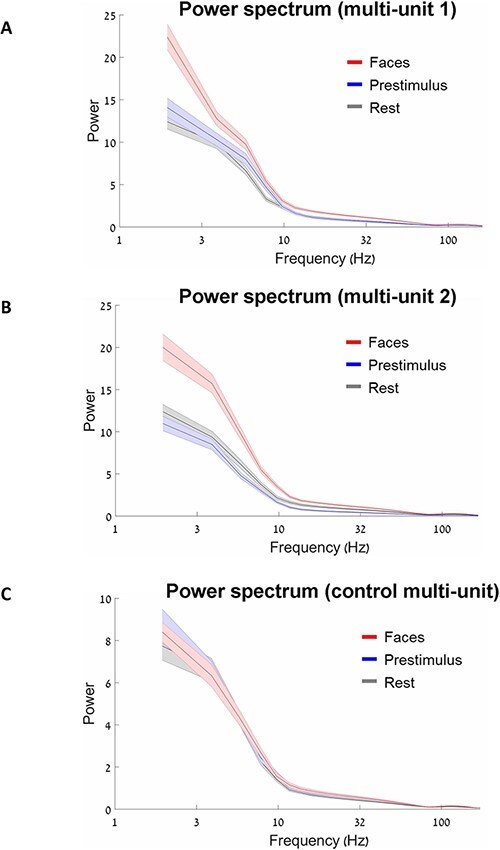
Comparison of power spectral density between conditions. (A) Results in multi-unit 1. (B) Results in multi-unit 2. (C) Results in control multi-unit. Note, a major difference in power spectrum across the frequencies between stimulus-evoked face and both spontaneous conditions in multi-units 1 and 2 but not in control multi-unit. Error shadows reflect standard error of mean (SEM).

**Figure 6.**
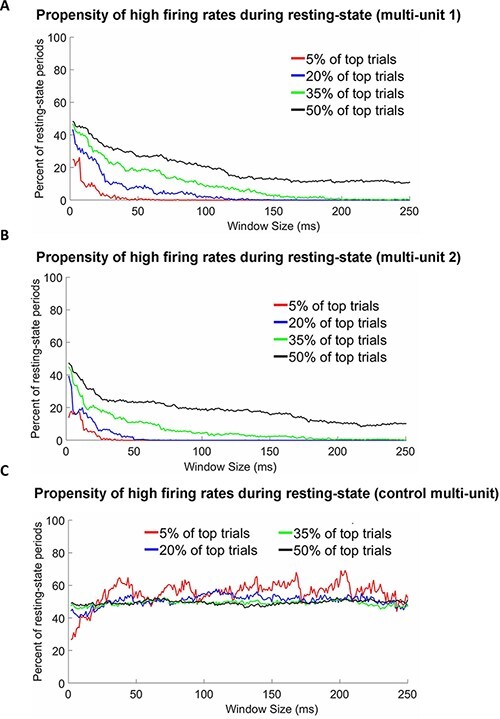
The percentage of windows taken from spontaneous activity that have a higher firing rate than windows taken from selective stimulus-evoked periods in the faces condition with different proportions of the top firing values (i.e. top 5%, top 20%, top 35%, and all 50% of windows). (A) Results in multi-unit 1. (B) Results in multi-unit 2. (C) Results in the control multi-unit. Note that for multi-units 1 and 2, for time windows with a duration of 200 ms and longer, no resting-state windows were within 35% of the top firing windows. In addition, for the very short time windows (20 ms and below), some resting-state windows were within 5% of the top firing windows. In contrast, for the control multi-unit, the top percentages of the highest firing rates fluctuate ∼50% (regardless of the percentage threshold used).

The entire stimulus-evoked experiment was 11 minutes and 23 seconds long. The recording of this session was split into two parts. The first part of the recording had an equal duration to that of the resting-state session (5 minutes and 50 seconds). Our main analyses (Figs. 3–6) were conducted using the data of the first part. The second part was comprised of the remaining data, which was allocated to be used to establish selectivity using independent data (Kriegeskorte *et al.* 2009) (Fig. 2). Additionally, this second part of the data was used to determine the point of beginning, peak, and end of modulation of the faces condition compared to baseline. The data of the stimulus-evoked experiment were split into epochs, with a baseline (i.e. prestimulus) period of 500 ms and a trial period of 1250 ms.

In order to compare the distribution of firing rates during the faces condition, the prestimulus activity, and the resting state (Fig. 4), instantaneous firing rates were calculated by smoothing the binary spike train with a Gaussian kernel with a full width at half maximum (FWHM) of 80 ms [in accordance with previous similar analysis (Nir *et al.* 2008)]. The firing rates for the faces condition were calculated for the periods of significant modulation compared to baseline. Beginning and end points of these periods were obtained using independent data (see above). To obtain the relative proportion of each histogram bin, the histogram was normalized for each condition, based on the number of instantaneous firing rates extracted.

Comparison of power spectral density between conditions (Fig. 5) was conducted using Fast Fourier Transform (Matlab fft function). Qualitatively similar results were obtained when the power spectrum was calculated using multitaper technique (mtspectrumc chronux toolbox function). The spectrum was calculated for each trial of each condition and then averaged (for similar approach, see Mazzoni *et al.* 2008). Segment duration for all conditions was set to 500 ms to match the prestimulus duration. In the faces condition, the onset of the segment was the time of peak modulation for that unit relative to the baseline. The peak value for each multi-unit was calculated using independent data (see above). For the resting state, random periods were sampled (the results were the same independent of the samples used).

The analysis presented in Fig. 6 aimed to examine the durations of periods of resting-state activity that have higher firing rates compared to the stimulus-evoked response. This analysis has five steps. First, a duration of the time window to be used was defined. Throughout, we used lengths from 2 to 250 ms. Second, windows of this size were selected from the binary spike train for each of the faces stimulus-evoked trials (*n* = 70) and an equal number of resting-state windows. Third, we calculated the sum of spikes for each window, resulting in a vector of firing rates. This resulted in 70 (faces stimulus-evoked) + 70 (resting-state) = 140 values. Fourth, this vector was ranked in descending order. Fifth, we calculated the percentage of resting-state windows in the predefined percentiles of top ranked values (e.g. for the 70 windows with the highest firing rates for the 50% percentile, see below). Values on Y axis in Fig. 6 reflect the resulting percentage of resting-state windows. The onset of the time windows (regardless of the window’s length) for the stimulus-evoked response condition was the time of peak modulation for the faces condition for that unit relative to the baseline. The peak value for each multi-unit was calculated using independent data (see above). The onsets of time windows in the resting-state data were selected randomly. In order to have an equal number of windows for the stimulus-evoked response and the resting-state data, we bootstrapped the resting-state windows by repeating the analysis 1000 times and only then averaging the results. In our analysis, we examined four different percentiles of the top-firing rates (i.e. sum of spikes within window). We examined the percentage of the resting-state windows among the top 5%, top 20%, top 35%, and top 50% of values. For example, for the top 5%, we asked what percentage of resting-state windows were within the top 7 (140 × 5% = 7) windows with the highest firing rates. The analysis comparing faces stimulus-evoked activity and prestimulus activity was conducted in the same way but without boostrapping.

## Results

During the resting-state session (5 minutes and 50 seconds), patients were asked to keep their eyes closed, not to think about anything in particular, and to not imagine anything. The stimulus-evoked experiment included static images of familiar (i.e. famous) and unfamiliar faces. In addition, the experiment included images of familiar and unfamiliar city views (referred to below as “scenes”). The scenes condition was used only to establish face selectivity and was not used in our main analyses. Each stimulus was preceded by a fixation period (referred to below as “prestimulus activity”). The behavioral task was to indicate whether the image was familiar or not (see the “Methods” section for more details). The performance of the patient with face-selective multi-units was almost perfect for faces (familiar faces = 100%, unfamiliar faces = 95%) but worse for scenes (familiar scenes = 95%, unfamiliar scenes = 22%). The low performance for unfamiliar scenes was because the patient mistakenly thought that the scene should be indicated as “familiar” even if it only resembles a familiar scene. The performance of the control patient was high for both faces (familiar faces = 99%, unfamiliar faces = 85%) and scenes (familiar scenes = 94%, unfamiliar scenes = 93%). As we were interested in the stimulus-selective response in general, we combined familiar and unfamiliar stimuli, resulting in two conditions: “faces” and “scenes”.

The averaged stimulus-evoked responses (based on the second part of the stimulus-evoked experiment) are shown in Fig. 2. In multi-units 1 and 2, we see that faces elicited strong stimulus-evoked modulation compared to baseline. Additionally, faces elicited a much higher stimulus-evoked response compared to the scenes condition (i.e. face-selectivity). Statistically, the activity in the faces condition was beyond the baseline in multi-unit 1 [the period between 84 and 751 ms after stimulus onset, *P* < 0.001, two-tailed nonparametric cluster-based permutations (Maris and Oostenveld 2007)] and in multi-unit 2 (the period between 91 and 768 ms after stimulus onset, *P* < 0.001). The peak values of the faces condition relative to baseline (i.e. the highest t-values) were at 176 ms from stimulus onset (multi-unit 1) and at 173 ms from stimulus onset (multi-unit 2). In addition to beyond baseline responses in the faces condition, in multi-unit 2, the activity increased beyond the baseline in the scenes condition (the period between 131 and 483 ms after stimulus onset, *P* < 0.001; peak value at 330 ms from stimulus onset). Comparing the faces and scenes conditions revealed a face-selective response in multi-unit 1 ([99 ms:1000 ms period, *P* < 0.001) and multi-unit 2 ([107 ms:733 ms period, *P* < 0.001). Overall, these results establish high face selectivity of the two recorded multi-units. In the control multi-unit (Fig. 2C), the were no periods of beyond-baseline modulation for neither the faces nor the scenes condition. In addition, there were no periods of higher activity for faces compared to scenes (i.e. no face-selectivity).

Next, we proceed to the main goal of the present investigation, comparing face-selective stimulus-evoked and spontaneous activity (i.e. resting-state and prestimulus activity). Note that all of the following analyses that included stimulus-evoked conditions were conducted using data that were independent from those used to establish selectivity (see above). In Fig. 3, we show representative examples of instantaneous firing rate traces of the stimulus-evoked experiment (Fig. 3A) and resting-state session (Fig. 3B), recorded from face-selective multi-unit 1. Data were smoothed with a Gaussian window of FWHM of 80 ms (Nir *et al.* 2008). This figure illustrates that in many cases faces elicited strong firing rate modulation at the level of single stimuli. The firing rates preceding the stimulus and during the resting state were weaker, but interestingly, there were also occasional, relatively short periods of strong firing rates comparable to those of the face-evoked activity.

To take a quantitative look at the data, we first compared the average firing rates of the segments of face-evoked activity, prestimulus activity, and the resting-state activity. The segments of stimulus-evoked activity were taken from the periods (in time) of the beyond-baseline modulation of the faces condition calculated on independent data (see above). We found that for multi-units 1 and 2, the average stimulus-evoked firing rate in the faces condition was much higher compared to resting-state activity and prestimulus activity periods: multi-unit 1 faces mean = 48.6 spikes/second, SEM = 0.15; multi-unit 1 fixation mean = 15.9 spikes/second, SEM = 0.09; multi-unit 1 resting-state mean = 14.95 spikes/second, SEM = 0.03; multi-unit 2 faces mean = 48.21 spikes/second, SEM = 0.16; multi-unit 2 fixation mean = 14.7 spikes/second, SEM = 0.09; multi-unit 2 resting-state mean = 10.38 spikes/second, SEM = 0.023. In contrast, in the control multi-unit, there was no major difference between the faces condition, the resting-state session, and the prestimulus activity: faces mean = 13.6 spikes/second, SEM = 0.06; prestimulus activity mean = 11.79 spikes/second, SEM = 0.06; resting-state mean = 12.5 spikes/second, SEM = 0.02.

Next, to explore the firing rate profile in more detail, we compared the distribution of instantaneous firing rates of the three conditions (i.e. face-selective evoked response, prestimulus activity, and the resting-state activity). The results are shown in Fig. 4. For multi-units 1 and 2, we found a reversed pattern when comparing the faces condition to the resting-state and prestimulus activities: the low firing rates were abundant in the resting-state (gray bars) and prestimulus activities (blue) but were uncommon in the faces condition (red bars); in contrast, the high firing rates were abundant in the faces condition but were almost absent in the resting-state and prestimulus activities. In contrast, in the control multi-unit, there was no major difference between the patterns of activity of the three conditions.

At the next stage, we compared the power spectral density between conditions. The power spectrum was calculated for single trials, and then, the result was averaged. In multi-units 1 and 2 (Fig. 5A and B), we can see the major differences in power spectrum across the frequencies between stimulus-evoked face condition and two spontaneous conditions. In contrast, for the control multi-unit (Fig. 5C), we do not see any differences between conditions in power spectrum.

We have already established that the selective (i.e. face-selective) stimulus-evoked response is characterized by a higher firing rate compared to resting-state activity (Fig. 4). But in the illustrative example in Fig. 3, we observed that periods with high firing rates could be also found during spontaneous activity. In the quantitative analyses that follow, we explored how probable it was that the firing rate during the resting state was higher than during face condition. In contrast to the previous analyses, the present analysis was conducted using binary spike train data (i.e. the data without Gaussian smoothing), permitting us to investigate how the length of time over which the activity is examined influences the results. In the analysis presented below, we compare stimulus-evoked face-selective responses with resting-state activity. Comparing stimulus-evoked face-selective responses and prestimulus activity produced qualitatively similar results. The analysis was conducted for a time window that varied in length from 2 to 250 ms. For each of the stimulus-evoked trials (70 trials, in the faces condition) and for an equal number of resting-state time windows, we calculated the sum of spikes over a given window. This resulted in a vector of 140 values. Then, we calculated the percentage of the resting-state values among predefined percentage of the highest values (values on Y axis in Fig. 6). Specifically, we examined the percentage of resting-state windows among the top 5%, 20%, 35% and 50% of windows with the highest firing rates. For more details, see the ‘Methods’ section.

The results of the analysis are shown in Fig. 6. For multi-units 1 and 2, we observed the following: (i) the longer the time-window, the less likely it was to encounter firing rates from the resting-state session that were comparable to the face-selective response; (ii) proportion of resting-state time windows with a firing-rate comparable to that of the selective stimulus-evoked response depended on the percentage threshold used. Critically, with short windows, even for the top 5% of windows (red trace), some resting-state windows were comparable to the evoked face-selective firing rate; (iii) and for the longer time windows (e.g. longer than ∼50−100 ms for 20% threshold and below or longer than ∼150−200 ms for thresholds 35%−50%), there were few resting-state time windows with a firing rate comparable to that of the selective stimulus-evoked response. In contrast, for the control multi-unit (Fig. 6C), a completely different pattern emerged, such that the percent of resting-state windows with higher firing rates fluctuates around 50% (regardless of the percentage threshold used).

Finally, a possible concern is that during the stimulus-evoked experiment, the subject was not attentive to all stimuli, resulting in a lower evoked response for these “missed” trials. But this is likely not the case because the behavioral performance of the patient in the faces condition was almost perfect. Yet, to rule out even a minimal potential confound, we repeated all our analyses while we excluded two face trials that were categorized incorrectly. The results from this repeated analysis were indistinguishable from those reported earlier. Thus, our results are unlikely to be explained by inattention to stimuli.

## Discussion

In the present investigation, we compared multi-unit face-selective stimulus-evoked and spontaneous responses in two face-selective multi-units in the human posterior fusiform gyrus. We found that on average, face-selective stimulus-evoked firing rates were much higher than spontaneous activity. However, examining activity over varying time windows revealed a more nuanced picture. Specifically, we found that when the activity was examined over a relatively short time window (e.g. 50 ms), many periods of spontaneous activity were characterized by high firing rates, comparable to stimulus-evoked firing. Our results indicate that conscious awareness of a stimulus might be associated with a sustained response in the selective region. Below, we discuss our findings in more detail.

Spontaneous (or ongoing) neural activity is one of the most enigmatic phenomena related to brain processing (Moutard *et al.* 2015; Raichle 2015). Spontaneous activity happens constantly and is responsible for most of the brain’s energy consumption (Raichle and Mintun 2006). Therefore, it is of primary importance to understand the role and functional principles of spontaneous activity. An important property of spontaneous activity is that its fluctuations are unconscious. Conversely, stimulus-evoked activity may or may not be associated with conscious percept. For example, according to the taxonomy proposed by Dehaene and colleagues (Dehaene *et al.* 2006), stimulus-evoked activity will be associated with conscious percept (in case of long-distance reverberation) or will not be associated with conscious percept (“preconscious” mode: in case of a local reverberation or “subliminal” mode: in case of no reverberation). Accordingly, here we asked how the stimulus-evoked neural response that is associated with conscious percept differs from the spontaneous response that is not associated with the conscious percept. In the past, this question has been only partially addressed, likely because of the lack of an appropriate experimental setup. That is, compared to several previous studies (Arieli *et al.* 1995; Nir *et al.* 2006, 2008), our experimental setup was particularly suitable for addressing this question because (i) the multi-unit recording that we used is a direct measure of neural activity, in contrast to fMRI which is an indirect measure (Logothetis *et al.* 2001); (ii) conducting the experiment with a human subject allows us to know definitively the conscious perception of the participant; and (iii) the recording in our study was conducted in the FFA—the high-level visual region that may directly contribute to conscious awareness of a specific content (i.e. a face) (Block 2007; Boly *et al.* 2017).

Our results can be divided into two parts. First, in line with the results of one earlier study that recorded activity in the auditory cortex of human patients (Nir *et al.* 2008), we found that when activity in the FFA was examined over relatively long temporal windows (100–200 ms and longer) or large temporal Gaussian smoothing (FWHM = 80 ms) was used, stimulus-evoked activity was much higher than spontaneous activity (both resting-state and prestimulus activities). Second, most interestingly, our finding was that when the activity was examined over relatively short windows (50 ms and smaller, depending on the threshold used), resting-state activity was often high and comparable to stimulus-evoked activity (Fig. 6). Critically, this result cannot be explained by low responses during stimulus-evoked trials because (i) we used the highest level of stimulus-evoked activity as the onset of our time-windows and (ii) when the window length was 20 ms and below, the windows of the spontaneous activity could be found within even the top 5% of the windows with the highest firing rates. Also note that during the stimulus-evoked experiment the participant attended to the stimuli, as was reflected by her excellent performance in the behavioral task. Taken together, our results indicate that the important difference between stimulus-evoked activity that is associated with a conscious percept and the spontaneous activity that is not associated with a conscious percept might be how long the activity is sustained. That being said, the present result cannot establish whether the stimulus-evoked sustained activity in the FFA directly contributes to conscious awareness (i.e. serves as a neural correlate of consciousness). Interestingly, while in the present study we tested spontaneous activity that was not associated with conscious content awareness, there has also been extensive research that examined stimulus-evoked activity that is not associated with conscious awareness about some content (Dehaene and Changeux 2011; van Gaal and Lamme 2011; Hesselmann 2013; Axelrod *et al.* 2015a). For example, subjectively invisible stimuli of faces elicit an evoked response in the FFA (Sterzer *et al.* 2008; Fahrenfort *et al.* 2012). From a theoretical point of view, it has been suggested that the main difference between unconscious and conscious evoked-activity might be that only the latter is reverberative and sustained (Dehaene and Naccache 2001; Dehaene *et al.* 2006; Lamme 2006). To this extent, our results point to potentially similar mechanisms of unconscious spontaneous and unconscious stimulus-evoked activity.

A potential concern that can be raised regarding our findings is whether the participant was engaged in facial imagery during the resting-state session. Indeed, a recent study that also recorded single- and multi-unit face-selective activity reported similarity in firing rate patterns during perception and imagery of a face (Khuvis *et al.* 2021). However, we think that facial imagery is unlikely a major factor that could explain our results. First, our participant got clear instructions before the resting-state session to not think about anything in particular, including to not be engaged in imagery. Second, neural activity that can be measured during imagery is by definition weaker compared to sensory stimuli (e.g. O’Craven and Kanwisher 2000). Accordingly, studies that examine neural activity for imagery of faces adopt special procedures to achieve reliable neural activity. For example, when visual stimuli are shown prior to the imagery stage, the participants are explicitly asked to remember fine facial details like unique color and facial expression (Khuvis *et al.* 2021). Another approach to facilitate imagery is to use only a few facial identities and to show visual stimuli immediately prior to the imagery task (Dijkstra *et al.* 2018). Thus, in our case, even if the participant had a brief, fleeting thought relating to a person, it is unlikely that this would have resulted in reliable neural activity in the FFA. Third, we showed that the resting-state and stimulus-evoked patterns of activity were distinct (i.e. a sustained response in the stimulus-evoked case and a brief response in the resting-state; see example in Fig. 3). Had imagery occurred during the resting-state session, the patterns of the activity during resting state would have appeared similar to the stimulus-evoked activity. Finally, according to our results (Fig. 6), for the window length of 50 ms, we found ∼25% of the resting-state windows with activity comparable to stimulus-evoked activity. Even if facial imagery occurred occasionally during the resting-state session, it is highly unlikely that it occurred so frequently (i.e. many dozens of times during a 6-minute period). Note that similar to the research of mind-wandering and self-generated cognition (Smallwood and Schooler 2015), there is no straightforward solution on how to establish the extent of face imagery during the 6-minute resting-state session. One possibility is to include a formal questionnaire after the resting-state session regarding potential imagery of a face (Diaz *et al.* 2013; Gorgolewski *et al.* 2014)—the procedure that was not implemented in our study. However, reliability of the responses in such a case can be questioned because the participant might not remember at the end of the session what was going through their mind throughout the entire period. Another possibility is to include thought probes throughout the resting-state session (e.g. Robertson *et al.* 1997; Axelrod *et al.* 2015b). However, while frequent probes might disrupt the spontaneous nature of a thought, infrequent probes suffer from the limitation we discussed above with regard to the questionnaire at the end of the session. Overall, there is an inherent difficulty to establish reliable imagery phenomenology in a spontaneous experiment. However, as we pointed out above, facial imagery was unlikely a strong factor impacting our results.

There have been two main directions in the investigation of spontaneous activity. The first is the so-called resting-state—a continuous session without any task (Raichle *et al.* 2001). In the field of consciousness research, resting-state spontaneous activity has been linked to and is usually investigated in the context of level or state of consciousness. In particular, spontaneous activity has been shown to vary across conscious states, such as anesthesia (Barttfeld *et al.* 2015; Huang *et al.* 2016), sleep (Tagliazucchi and Laufs 2014), or disorder of consciousness states (Cao *et al.* 2019; Huang *et al.* 2020). Theoretically, according to the Temporo-spatial Theory of Consciousness (TTC), the role of spontaneous activity is to integrate across space and time, thus creating a situation of a neural predisposition of consciousness (Northoff and Huang 2017; Northoff and Lamme 2020). Note, however, that the focus of the present work was stimulus conscious awareness but not conscious state; therefore, our results are not directly related to this body of research. The second research direction has been the influence and interaction of the spontaneous prestimulus activity on the subsequent stimulus-evoked activity (Sadaghiani *et al.* 2010). In a seminal study conducted more than a decade ago, Hesselmann and colleagues showed that higher prestimulus activity in the FFA increased the probability that the Rubin face-vase illusion stimulus is perceived as a face (Hesselmann *et al.* 2008b) (for related findings for other brain regions and modalities, see Hesselmann *et al.* 2008a; Sadaghiani *et al.* 2010). Since then, more studies demonstrated that prestimulus activity can shape various properties of stimulus-related activity (e.g. level of activation and trial-to-trial variability; Podvalny *et al.* 2019; Northoff and Lamme 2020). A major theoretical conceptualization of this phenomenon has been proposed by TTC, according to which the ongoing (spontaneous) activity and the stimulus-evoked response need to be integrated in order for the stimulus to become conscious (Northoff and Huang 2017; Northoff and Lamme 2020). In other words, if the stimulus “arrives” at the wrong phase of the ongoing wave, the stimulus might remain unconscious. To this extent, an additional important line of investigation has been to understand the interaction between spontaneous activity and stimulus-evoked activity. Specifically, it has been suggested that this interaction might be nonadditive (He 2013; Huang *et al.* 2015), while Huang and colleagues (Huang *et al.* 2015) also suggested that interaction between spontaneous and stimulus-evoked activity might depend on the phase of spontaneous activity. In our work, we compared the properties of the spontaneous (both resting-state and prestimulus activities) with stimulus-evoked activity. However, we did not address the question of interaction between prestimulus activity and stimulus activity because our experiment was not suited for that. That is, our experiment included images of 20 different facial identities (half of them familiar and half of them unfamiliar to the participant). Different facial identities might result in different neural activities in the FFA (Davidesco *et al.* 2013; Ghuman *et al.* 2014; Axelrod and Yovel 2015; Khuvis *et al.* 2021). In addition, familiarity of a face can also modulate the response in the FFA (Weibert and Andrews 2015; Axelrod *et al.* 2019). Such variability across trials in stimulus-evoked activity due to a difference in stimuli is obviously a serious confound when one investigates the interaction between prestimulus and stimulus activity. Note that the fact that different facial stimuli might have resulted in different responses did not limit interpretation of the findings presented in the paper. This is because we did not focus on individual trials but rather on the faces condition as a whole.

Finally, an evident limitation of the present study is that it was conducted using only two face-selective multi-units. Therefore, any potential generalization to a large neural population should be made with caution. Nevertheless, the rare setup we used gave us insight that could not be obtained in previous studies. To this extent, studies with one patient (Parvizi *et al.* 2012; Rosenbaum *et al.* 2014; Aminoff *et al.* 2016; Jonas *et al.* 2018; Liu *et al.* 2018; Pereira *et al.* 2021; Streese and Tranel 2021) or few units (Self *et al.* 2016) have traditionally been important in cognitive neuroscience. Thus, we believe that the present study constitutes a valuable contribution to the understanding of spontaneous activity and its relationship to stimulus-evoked activity.

## Summary

In the present study, we compared task-evoked activity that was associated with conscious awareness and spontaneous activity. We found that when activity was examined for the relatively long temporal windows (e.g. 100–200 ms), stimulus-evoked firing was much higher than in the resting-state activity. However, examination of the activity for the relatively short windows (e.g. 50 ms) revealed many occurrences of resting-state activity with the firing rates comparable to stimulus-evoked activity. Thus, how sustained a response is may differentiate between stimulus-evoked activity that is associated with conscious awareness and the spontaneous activity that is not associated with conscious perception.

## Data Availability

Data not publicly available. The data of the paper is clinical, patient data. Therefore, it cannot be shared.

## References

[R1] Aminoff EM , Li Y, Pyles JA et al. Associative hallucinations result from stimulating left ventromedial temporal cortex. *Cortex* 2016;83:139–44. 27533133 10.1016/j.cortex.2016.07.012 PMC5228589

[R2] Arieli A , Shoham D, Hildesheim R et al. Coherent spatiotemporal patterns of ongoing activity revealed by real-time optical imaging coupled with single-unit recording in the cat visual cortex. *J Neurophysiol* 1995;73:2072–93. 7623099 10.1152/jn.1995.73.5.2072

[R3] Arieli A , Sterkin A, Grinvald A et al. Dynamics of ongoing activity: explanation of the large variability in evoked cortical responses. *Science* 1996;273:1868–71. 8791593 10.1126/science.273.5283.1868

[R4] Axelrod V . Minimizing bugs in cognitive neuroscience programming. *Front Psychol* 2014;5. 10.3389/fpsyg.2014.01435 PMC4269119 25566120

[R5] Axelrod V , Bar M, Rees G. Exploring the unconscious using faces. *Trends Cogn Sci*2015a;19:35–45. 25481216 10.1016/j.tics.2014.11.003

[R6] Axelrod V , Rees G, Lavidor M et al. Increasing propensity to mind wander with transcranial direct current stimulation. *Proc Natl Acad Sci U S A*2015b;112:3314–9. 25691738 10.1073/pnas.1421435112 PMC4371998

[R7] Axelrod V , Rozier C, Malkinson TS et al. Face-selective neurons in the vicinity of the human fusiform face area. *Neurology* 2019;92:197–8. 30665911 10.1212/WNL.0000000000006806

[R8] Axelrod V , Yovel G. Successful decoding of famous faces in the Fusiform Face Area. *PLoS One* 2015;10:e0117126. 10.1371/journal.pone.0117126 PMC4340964 25714434

[R9] Babo-Rebelo M , Wolpert N, Adam C et al. Is the cardiac monitoring function related to the self in both the default network and right anterior insula? *Phil Trans R Soc B* 2016;371. 10.1098/rstb.2016.0004 PMC5062094 28080963

[R10] Barttfeld P , Uhrig L, Sitt JD et al. Signature of consciousness in the dynamics of resting-state brain activity. *Proc Natl Acad Sci U S A* 2015;112:887–92. 25561541 10.1073/pnas.1418031112 PMC4311826

[R11] Berger H . Über das elektroenkephalogramm des menschen. *Arch Psychiatr Nervenkr* 1929;87:527–70.

[R12] Biswal B , Zerrin Yetkin F, Haughton VM et al. Functional connectivity in the motor cortex of resting human brain using echo-planar mri. *Magn Reson Med* 1995;34:537–41. 8524021 10.1002/mrm.1910340409

[R13] Block N . Consciousness, accessibility, and the mesh between psychology and neuroscience. *Behav Brain Sci* 2007;30:481–99. 18366828 10.1017/S0140525X07002786

[R14] Boly M , Massimini M, Tsuchiya N et al. Are the neural correlates of consciousness in the front or in the back of the cerebral cortex? Clinical and neuroimaging evidence. *J Neurosci* 2017;37:9603–13. 28978697 10.1523/JNEUROSCI.3218-16.2017 PMC5628406

[R15] Brainard DH . The psychophysics toolbox. *Spat Vis* 1997;10:433–6. 9176952

[R16] Broday-Dvir R , Malach R. Resting-state fluctuations underlie free and creative verbal behaviors in the human brain. *Cereb Cortex* 2021;31:213–32. 32935840 10.1093/cercor/bhaa221

[R17] Bullmore E , Sporns O. Complex brain networks: graph theoretical analysis of structural and functional systems. *Nat Rev Neurosci* 2009;10:186–98. 19190637 10.1038/nrn2575

[R18] Cao B , Chen Y, Yu R et al. Abnormal dynamic properties of functional connectivity in disorders of consciousness. *NeuroImage Clin* 2019;24. 10.1016/j.nicl.2019.102071 PMC6881656 31795053

[R19] Chammat M , El Karoui I, Allali S et al. Cognitive dissonance resolution depends on episodic memory. *Sci Rep* 2017;7. 10.1038/srep41320 PMC5256105 28112261

[R20] Corlier J , Valderrama M, Navarrete M et al. Voluntary control of intracortical oscillations for reconfiguration of network activity. *Sci Rep* 2016;6. 10.1038/srep36255 PMC5093688 27808225

[R21] Damoiseaux JS , Rombouts SARB, Barkhof F et al. Consistent resting-state networks across healthy subjects. *Proc Natl Acad Sci U S A* 2006;103:13848–53. 16945915 10.1073/pnas.0601417103 PMC1564249

[R22] Davidesco I , Zion-Golumbic E, Bickel S et al. Exemplar selectivity reflects perceptual similarities in the human fusiform cortex. *Cereb Cortex* 2013;24:1879–93. 23438448 10.1093/cercor/bht038 PMC4051894

[R23] Dehaene S , Changeux J-P. Experimental and theoretical approaches to conscious processing. *Neuron* 2011;70:200–27. 21521609 10.1016/j.neuron.2011.03.018

[R24] Dehaene S , Changeux J-P, Naccache L et al. Conscious, preconscious, and subliminal processing: a testable taxonomy. *Trends Cogn Sci* 2006;10:204–11. 16603406 10.1016/j.tics.2006.03.007

[R25] Dehaene S , Naccache L. Towards a cognitive neuroscience of consciousness: basic evidence and a workspace framework. *Cognition* 2001;79:1–37. 11164022 10.1016/s0010-0277(00)00123-2

[R0025a] Diaz BA , Van Der Sluis S, Moens S et al. The Amsterdam resting-state questionnaire reveals multiple phenotypes of resting-state cognition. *Front Hum Neurosci* 2013;7. 10.3389/fnhum.2013.00446 PMC3737475 23964225

[R26] Dijkstra N , Mostert P, de Lange FP et al. Differential temporal dynamics during visual imagery and perception. *eLife* 2018;7. 10.7554/eLife.33904 PMC5973830 29807570

[R27] El Karoui I , King J-R, Sitt J et al. Event-related potential, time-frequency, and functional connectivity facets of local and global auditory novelty processing: an intracranial study in humans. *Cereb Cortex* 2014;25:4203–12. 24969472 10.1093/cercor/bhu143 PMC5635961

[R28] Fahrenfort JJ , Snijders TM, Heinen K et al. Neuronal integration in visual cortex elevates face category tuning to conscious face perception. *Proc Natl Acad Sci U S A* 2012;109:21504–9. 23236162 10.1073/pnas.1207414110 PMC3535630

[R29] Fox MD , Corbetta M, Snyder AZ et al. Spontaneous neuronal activity distinguishes human dorsal and ventral attention systems. *Proc Natl Acad Sci U S A* 2006;103:10046–51. 16788060 10.1073/pnas.0604187103 PMC1480402

[R30] Friston K . A theory of cortical responses. *Philos Trans R Soc Lond B Biol Sci* 2005;360:815–36. 15937014 10.1098/rstb.2005.1622 PMC1569488

[R0030a] Ghuman AS , Brunet NM, Li Y et al. Dynamic encoding of face information in the human fusiform gyrus. *Nat Commun* 2014;5. 10.1038/ncomms6672 PMC4339092 25482825

[R0031a] Gorgolewski KJ , Lurie D, Urchs S et al. A correspondence between individual differences in the brain’s intrinsic functional architecture and the content and form of self-generated thoughts. *PLoS One* 2014;9. 10.1371/journal.pone.0097176 PMC4019564 24824880

[R31] Hahamy A , Wilf M, Rosin B et al. How do the blind ‘see’? The role of spontaneous brain activity in self-generated perception. *Brain* 2020;144:340–53. 10.1093/brain/awaa384 PMC7880672 33367630

[R32] He BJ . Spontaneous and task-evoked brain activity negatively interact. *J Neurosci* 2013;33:4672–82. 23486941 10.1523/JNEUROSCI.2922-12.2013 PMC3637953

[R33] He BJ , Raichle ME. The fMRI signal, slow cortical potential and consciousness. *Trends Cogn Sci* 2009;13:302–9. 19535283 10.1016/j.tics.2009.04.004 PMC2855786

[R34] Hesselmann G . Dissecting visual awareness with fMRI. *Neuroscientist* 2013;19:495–508. 23599237 10.1177/1073858413485988

[R35] Hesselmann G , Kell CA, Eger E et al. Spontaneous local variations in ongoing neural activity bias perceptual decisions. *Proc Natl Acad Sci U S A*2008b;105:10984–9. 18664576 10.1073/pnas.0712043105 PMC2504783

[R36] Hesselmann G , Kell CA, Kleinschmidt A. Ongoing activity fluctuations in hMT+ bias the perception of coherent visual motion. *J Neurosci*2008a;28:14481–5. 19118182 10.1523/JNEUROSCI.4398-08.2008 PMC6671252

[R37] Huang Z , Zhang J, Longtin A et al. Is there a nonadditive interaction between spontaneous and evoked activity? Phase-dependence and its relation to the temporal structure of scale-free brain activity. *Cereb Cortex* 2015;27:1037–59. 10.1093/cercor/bhv288 26643354

[R38] Huang Z , Zhang J, Wu J et al. Temporal circuit of macroscale dynamic brain activity supports human consciousness. *Sci Adv* 2020;6. 10.1126/sciadv.aaz0087 PMC7065875 32195349

[R39] Huang Z , Zhang J, Wu J et al. Decoupled temporal variability and signal synchronization of spontaneous brain activity in loss of consciousness: an fMRI study in anesthesia. *NeuroImage* 2016;124:693–703. 26343319 10.1016/j.neuroimage.2015.08.062

[R0039a] Jonas J , Brissart H, Hossu G et al. A face identity hallucination (palinopsia) generated by intracerebral stimulation of the face-selective right lateral fusiform cortex. *Cortex* 2018;99:296–310. 29306709 10.1016/j.cortex.2017.11.022

[R40] Kanwisher N . The quest for the FFA and where it led. *J Neurosci* 2017;37:1056–61. 28148806 10.1523/JNEUROSCI.1706-16.2016 PMC5296790

[R41] Khuvis S , Yeagle EM, Norman Y et al. Face-selective units in human ventral temporal cortex reactivate during free recall. *J Neurosci* 2021;41:3386–99. 33431634 10.1523/JNEUROSCI.2918-19.2020 PMC8051680

[R42] Kornblith S , Quiroga RQ, Koch C et al. Persistent single-neuron activity during working memory in the human medial temporal lobe. *Curr Biol* 2017;27:1026–32. 28318972 10.1016/j.cub.2017.02.013 PMC5510887

[R43] Kriegeskorte N , Simmons WK, Bellgowan PSF et al. Circular analysis in systems neuroscience: the dangers of double dipping. *Nat Neurosci* 2009;12:535–40. 19396166 10.1038/nn.2303 PMC2841687

[R44] Lamme VAF . Towards a true neural stance on consciousness. *Trends Cogn Sci* 2006;10:494–501. 16997611 10.1016/j.tics.2006.09.001

[R45] Liu TT , Nestor A, Vida MD et al. Successful reorganization of category-selective visual cortex following occipito-temporal lobectomy in childhood. *Cell Rep* 2018;24:1113–22.e1116. 30067969 10.1016/j.celrep.2018.06.099 PMC6152879

[R46] Logothetis NK , Pauls J, Augath M et al. Neurophysiological investigation of the basis of the fMRI signal. *Nature* 2001;412:150–7. 11449264 10.1038/35084005

[R47] Lurie DJ , Kessler D, Bassett DS et al. Questions and controversies in the study of time-varying functional connectivity in resting fMRI. *Netw Neurosci* 2019;4:30–69. 10.1162/netn_a_00116 PMC7006871 32043043

[R48] Mantini D , Perrucci MG, Del Gratta C et al. Electrophysiological signatures of resting state networks in the human brain. *Proc Natl Acad Sci U S A* 2007;104:13170–5. 17670949 10.1073/pnas.0700668104 PMC1941820

[R49] Maris E , Oostenveld R. Nonparametric statistical testing of EEG-and MEG-data. *J Neurosci Methods* 2007;164:177–90. 17517438 10.1016/j.jneumeth.2007.03.024

[R50] Marron TR , Berant E, Axelrod V et al. Spontaneous cognition and its relationship to human creativity: a functional connectivity study involving a chain free association task. *NeuroImage* 2020;220. 10.1016/j.neuroimage.2020.117064 32574810

[R51] Mashour GA , Roelfsema P, Changeux J-P et al. Conscious processing and the global neuronal workspace hypothesis. *Neuron* 2020;105:776–98. 32135090 10.1016/j.neuron.2020.01.026 PMC8770991

[R52] Mazzoni A , Panzeri S, Logothetis NK et al. Encoding of naturalistic stimuli by local field potential spectra in networks of excitatory and inhibitory neurons. *PLoS Comput Biol* 2008;4. 10.1371/journal.pcbi.1000239 PMC2585056 19079571

[R53] Mitra P . *Observed Brain Dynamics*. USA: Oxford University Press, 2007.

[R54] Moutard C , Dehaene S, Malach R. Spontaneous fluctuations and non-linear ignitions: two dynamic faces of cortical recurrent loops. *Neuron* 2015;88:194–206. 26447581 10.1016/j.neuron.2015.09.018

[R55] Nir Y , Hasson U, Levy I et al. Widespread functional connectivity and fMRI fluctuations in human visual cortex in the absence of visual stimulation. *NeuroImage* 2006;30:1313–24. 16413791 10.1016/j.neuroimage.2005.11.018

[R56] Nir Y , Mukamel R, Dinstein I et al. Interhemispheric correlations of slow spontaneous neuronal fluctuations revealed in human sensory cortex. *Nat Neurosci* 2008;11:1100–8. 19160509 10.1038/nn.2177 PMC2642673

[R57] Northoff G . What the brain’s intrinsic activity can tell us about consciousness? A tri-dimensional view. *Neurosci Biobehav Rev* 2013;37:726–38. 23253946 10.1016/j.neubiorev.2012.12.004

[R58] Northoff G , Huang Z. How do the brain’s time and space mediate consciousness and its different dimensions? Temporo-spatial theory of consciousness (TTC). *Neurosci Biobehav Rev* 2017;80:630–45. 28760626 10.1016/j.neubiorev.2017.07.013

[R59] Northoff G , Lamme V. Neural signs and mechanisms of consciousness: is there a potential convergence of theories of consciousness in sight? *Neurosci Biobehav Rev* 2020;118:568–87. 32783969 10.1016/j.neubiorev.2020.07.019

[R60] O’Craven KM , Kanwisher N. Mental imagery of faces and places activates corresponding stimulus-specific brain regions. *J Cogn Neurosci* 2000;12:1013–23. 11177421 10.1162/08989290051137549

[R61] Oostenveld R , Fries P, Maris E et al. FieldTrip: open source software for advanced analysis of MEG, EEG, and invasive electrophysiological data. *Comput Intell Neurosci* 2011;2011. 10.1155/2011/156869 PMC3021840 21253357

[R62] Parvizi J , Jacques C, Foster BL et al. Electrical stimulation of human fusiform face-selective regions distorts face perception. *J Neurosci* 2012;32:14915–20. 23100414 10.1523/JNEUROSCI.2609-12.2012 PMC3517886

[R63] Pereira M , Megevand P, Tan MX et al. Evidence accumulation relates to perceptual consciousness and monitoring. *Nat Commun* 2021;12:1–11. 34059682 10.1038/s41467-021-23540-y PMC8166835

[R64] Podvalny E , Flounders MW, King LE et al. A dual role of prestimulus spontaneous neural activity in visual object recognition. *Nat Commun* 2019;10:1–13. 31477706 10.1038/s41467-019-11877-4 PMC6718405

[R65] Quiroga RQ , Nadasdy Z, Ben-Shaul Y. Unsupervised spike detection and sorting with wavelets and superparamagnetic clustering. *Neural Comput* 2004;16:1661–87. 15228749 10.1162/089976604774201631

[R66] Quiroga RQ , Reddy L, Kreiman G et al. Invariant visual representation by single neurons in the human brain. *Nature* 2005;435:1102–7. 15973409 10.1038/nature03687

[R67] Raichle ME . The brain’s default mode network. *Annu Rev Neurosci* 2015;38:433–47. 25938726 10.1146/annurev-neuro-071013-014030

[R68] Raichle ME , MacLeod AM, Snyder AZ et al. A default mode of brain function. *Proc Natl Acad Sci U S A* 2001;98:676–82. 11209064 10.1073/pnas.98.2.676 PMC14647

[R69] Raichle ME , Mintun MA. Brain work and brain imaging. *Annu Rev Neurosci* 2006;29:449–76. 16776593 10.1146/annurev.neuro.29.051605.112819

[R70] Reddy L , Poncet M, Self MW et al. Learning of anticipatory responses in single neurons of the human medial temporal lobe. *Nat Commun* 2015;6. 10.1038/ncomms9556 PMC4617602 26449885

[R71] Robertson IH , Manly T, Andrade J et al. Oops!’: performance correlates of everyday attentional failures in traumatic brain injured and normal subjects. *Neuropsychologia* 1997;35:747–58. 9204482 10.1016/s0028-3932(97)00015-8

[R72] Rosenbaum RS , Gilboa A, Moscovitch M. Case studies continue to illuminate the cognitive neuroscience of memory. *Ann N Y Acad Sci* 2014;1316:105–33. 24871381 10.1111/nyas.12467

[R73] Sadaghiani S , Hesselmann G, Friston KJ et al. The relation of ongoing brain activity, evoked neural responses, and cognition. *Front Syst Neurosci* 2010;4. 10.3389/fnsys.2010.00020 PMC2903187 20631840

[R74] Schurger A , Sitt JD, Dehaene S. An accumulator model for spontaneous neural activity prior to self-initiated movement. *Proc Natl Acad Sci U S A* 2012;109:E2904–13. 22869750 10.1073/pnas.1210467109 PMC3479453

[R75] Self MW , Peters JC, Possel JK et al. The effects of context and attention on spiking activity in human early visual cortex. *PLoS Biol* 2016;14. 10.1371/journal.pbio.1002420 PMC4807817 27015604

[R76] Smallwood J , Schooler JW. The science of mind wandering: empirically navigating the stream of consciousness. *Annu Rev Psychol* 2015;66:487–518. 25293689 10.1146/annurev-psych-010814-015331

[R77] Sterzer P , Haynes J-D, Rees G. Fine-scale activity patterns in high-level visual areas encode the category of invisible objects. *J Vis* 2008;8:1–12. 10.1167/8.15.10 19146294

[R78] Streese CD , Tranel D. Combined lesion-deficit and fMRI approaches in single-case studies: unique contributions to cognitive neuroscience. *Curr Opin Behav Sci* 2021;40:58–63. 33709012 10.1016/j.cobeha.2021.01.004 PMC7943030

[R79] Tagliazucchi E , Laufs H. Decoding wakefulness levels from typical fMRI resting-state data reveals reliable drifts between wakefulness and sleep. *Neuron* 2014;82:695–708. 24811386 10.1016/j.neuron.2014.03.020

[R80] Tsodyks M , Kenet T, Grinvald A et al. Linking spontaneous activity of single cortical neurons and the underlying functional architecture. *Science* 1999;286:1943–6. 10583955 10.1126/science.286.5446.1943

[R81] van Gaal S , Lamme VAF. Unconscious high-level information processing: implication for neurobiological theories of consciousness. *Neuroscientist* 2011;18:287–301. 21628675 10.1177/1073858411404079

[R82] Watanabe M , Cheng K, Murayama Y et al. Attention but not awareness modulates the BOLD signal in the human V1 during binocular suppression. *Science* 2011;334:829–31. 22076381 10.1126/science.1203161

[R83] Weibert K , Andrews TJ. Activity in the right fusiform face area predicts the behavioural advantage for the perception of familiar faces. *Neuropsychologia* 2015;75:588–96. 26187507 10.1016/j.neuropsychologia.2015.07.015

[R84] Yeo BT , Krienen FM, Sepulcre J et al. The organization of the human cerebral cortex estimated by intrinsic functional connectivity. *J Neurophysiol* 2011;106:1125–65. 21653723 10.1152/jn.00338.2011 PMC3174820

